# Attenuating Atherosclerosis through Inhibition of the NF-*κ*B/NLRP3/IL-1*β* Pathway-Mediated Pyroptosis in Vascular Smooth Muscle Cells (VSMCs)

**DOI:** 10.1155/2024/1506083

**Published:** 2024-03-25

**Authors:** Shihuan Li, Qingjie Li, Qiaofeng Zhou, Suqin Li, Siqi Wang, Qing Yao, Changhan Ouyang, Chao Liu, Mincai Li

**Affiliations:** ^1^College of Medicine, Hubei University of Science and Technology, Xianning 437100, China; ^2^Institute of Medicine, Hubei Key Laboratory of Diabetes, Hubei University of Science and Technology, Xianning, China; ^3^School of Basic Medical Science, Hubei University of Science and Technology, Xianning, China

## Abstract

**Objective:**

We investigated the effects of resveratrol (Res) and MCC950 on the pyroptosis of vascular smooth muscle cells (VSMCs) and the potential pathway.

**Methods and Results:**

Compared with the control (Con) group, the atherosclerosis (AS) group showed calcified nodules, which suggested that the calcification medium induced the calcification of VSMCs. VSMCs showed proliferative activity and significantly attenuated calcification under treatment with 10 *μ*mol/L Res. The calcium salt was detected by alizarin red S staining. Res and MCC950 downregulated the calcification, inflammatory, pyroptosis, and transcription factor-related indicators all decreased by RT-qPCR with Western blot and immunofluorescence. The results showed that Res and MCC950 refrained the calcification of VSMCs and that Res has a better effect than MCC950. Plaques and calcium salt deposits were present in the carotid region in the control group. More calcium salt deposits were evident in the plaques of the Par group by HE staining and alizarin red S staining. The calcification indexes BMP2, Runx2, and related indexes declined by immunofluorescence, which showed parthenolide-inhibited AS. The related protein expressions were consistent with the expression of the cell experiments.

**Conclusion:**

Our data demonstrated that inflammatory response and pyroptosis exacerbate AS and unravel the link between VSMCs and the progression of AS lesions. Res and MCC950 inhibited the calcification of VSMCs by regulating NF-*κ*B/NLRP3/IL-1*β* signaling axis. P53 can exacerbate the AS lesions by acting on NLRP3 inflammasome and pyroptosis. Our findings supported the clinical applications of Res and MCC950 in VSMCs individuals to counteract pyroptosis and AS, and P53 inhibitors also can be a potential treatment for AS.

## 1. Introduction

As the quality of life improves, the incidence of AS increases every year, which is a serious threat to human health. AS reduced the elasticity of arterial vessels and increased brittleness, leading to cerebral infarction and coronary heart disease [1]. AS is closely associated with lipid, inflammatory cell infiltration, vascular calcification, and calcium-phosphate complexes deposited in the vasculature [2]. Diabetes, hypertension, and chronic kidney disease can induce phenotypic transformation of VSMCs, inflammatory response, and pyroptosis, which activated the NF-*κ*B signaling pathway [3] and promoted the development of AS.

AS is a chronic inflammatory disease that triggers an immunoinflammatory response, oxidative stress, and pattern receptor recognition that identified damage-associated molecular patterns (DAMPs) and pathogen-associated molecular patterns (PAMPs), ultimately leading to inflammatory reaction. The NLRP3 inflammasome is a member of the nucleotide-binding oligomerization domain-like receptors (NLRs) that play an essential role in AS by mediating pyroptosis. The NLRP3 inflammasome consists of the NOD-like receptor NLRP3, apoptosis-associated speck-like protein containing CARD (ASC), and pro-Caspase-1. NLRP3 is classified into three domains: PYD, NACHT, and LRR. The activity of NLRP3 can be affected by phosphorylation, ubiquitination, and nitrosylation, while the PYD residue structural domain, NOD structural domain, and LRR structural domain can affect its phosphorylation [4]. The activated LRR region inhibits NLRP3 inflammasome activation through NEK7-NLRP3 complex with NIMA-related kinase 7 (NEK7), which plays an important role in AS, diabetes, Alzheimer's disease, and inflammatory bowel disease [5]. Reduced NLRP3 activity is not expected to be observed in vivo, as reactive oxygen species (ROS) and oxidative stress induce vascular gene expression and promote inflammatory responses leading to cell death.

Pyroptosis is an inflammatory form of gasdermin protein-mediated programmed cell death that releases massive amounts of inflammatory factors and manifests as a blistered plasma membrane morphology [6]. Additionally, the classical signaling pathway of pyroptosis involves the activation of Caspase-1 by the inflammasome, which then activates pro-IL-1*β* and pro-IL-18 to produce the proinflammatory factors IL-1*β* and IL-18 by protein hydrolysis. GSDMD then cleaves the N-terminal, which releases IL-1*β* and IL-18, inducing pyroptosis [7]. Studies have shown that the NLRP3 inflammasome-mediated inflammatory response and pyroptosis are highly correlated. The activation of pro-caspase-1 affects GSDMD protein by mediating the classical pathway of pyroptosis, leading to the oligomerization of the N-terminal structural domain, cell swelling, rupture, and release of cytoplasmic contents. Inflammasome also activates caspase-4, caspase-5, and caspase-11 to mediate the nonclassical pathway and cleaves GSDMD into GSDMD-NT and GSDMD-CT. The former binds to liposomal phospholipids to release IL-1*β* and induce pyroptosis [8]. Research has shown that nicotine combined with a high-fat diet in ApoE-/- mice induces silencing of the NLRP3 inflammasome in AS to inhibit endothelial pyroptosis [9]. Xu et al. found that nicotine also affects NF-*κ*B p65-mediated NLRP3 transcription to induce macrophage pyroptosis through HDAC6/NF-*κ*B/NLRP3 signaling axis [10]. Duewell [11] has reported that cholesterol crystals induced AS and promoted vascular endothelial pyroptosis by activating NLRP3 inflammasome. Vascular smooth muscle cells (VSMCs) accumulate from the arterial mesothelium into the intima, releasing extracellular matrix molecules, elastin, and interstitial collagen to form fibrous caps that cover plaques and promote AS. Therefore, NLRP3-mediated VSMCs focal death intervention can alleviate the lesions of AS.

Resveratrol (Res) is a natural polyphenol found in plants. It has several pharmacological effects, including anti-inflammatory, antioxidant, lipid regulator, and vasodilator [12]. Res inhibits the calcification of hVSMCs (human vascular smooth muscle cells) triggered by high levels of calcium and phosphorus. It also restrains the calcification induced by lipopolysaccharide in human lung epithelial cells [13]. Additionally, it reduces the expression of NLRP3, GSDMD, and caspase-1 [14]. MCC950 is a specific inhibitor of the inflammatory vesicles of NLRP3 [15]. It was reported that MCC950 and BHB inhibited the NLRP3 inflammasome, IL-1*β*, and IL-18 [16]. MCC950 has been found to reduce plaque in ApoE-/- mice induced by high-fat diets. It also suppressed NLRP3 inflammasome and pyroptosis of THP-1 macrophages abducted by ox-LDL [17–19]. However, there are few studies on the effects of Res and MCC950 on calcified VSMCs through the NF-*κ*B/NLRP3/IL-1*β* axis. Therefore, this study is aimed at investigating the effects of Res and MCC950 on the calcification of VSMCs via the NF-*κ*B/NLRP3/IL-1*β* signaling axis and the influences of P53 inhibitors on vascular plaques.

## 2. Materials and Methods

### 2.1. Materials

#### 2.1.1. Experimental Cells

Human vascular smooth muscle cell line (CRL-1999) was purchased from ATCC.

#### 2.1.2. Experimental Animals

Fourteen male SPF grade ApoE-/-male mice, 6-8 weeks old (22 ± 2 g) were purchased from Beijing Charles River Laboratory Animal Technology in an isolated environment. The study complied with the Guide for the Care and Use of Laboratory Animals. The animal study protocol was approved by the Institutional Animal Care and Use Committee of Hubei University of Science and Technology (permission number: SCXK(e)2020-0018).

#### 2.1.3. Main Reagents and Instruments

DMEM medium and fetal bovine serum (FBS) were purchased from Gibco, USA; Res (D10291) was purchased from Beijing Boaosen Biotechnology Co. Ltd; RNA Isolator Total RNA Extraction Reagent (R401-01), Reverse Transcription Kit HiScript III RT Super Mix for qPCR (R323), and Fluorescence Quantification Kit Ace Q Universal SYBR qPCR Master Mix (Q511-02) were purchased from Novozymes Biologicals; FITC-labeled goat anti-rabbit lgG (GB22303) and DAPI staining reagent (G1012) were obtained from Wuhan Seville Biotechnology Co. Ltd; IL-1*β* (A16288), IL-18 (A16737), and GSDMD (A18281) were purchased from ABclonal; BMP2 (sc-137087), Runx2 (sc-390351), NF-KBp65 (sc-8008) goat anti-rabbit lgG, and goat anti-mouse lgG were purchased from Santa Cruz, USA.

### 2.2. Experimental Methods

#### 2.2.1. Cellular Experiments

*(1) CCK-8 Assay for Cell Viability*. To detect cell proliferation by the CCK8 method, cultured VSMCs were logarithmically harvested and counted by trypsin digestion, inoculated at 1000 cells per well in a 96-well plate, and when the cells had grown to 70%, 5 replicate wells were set up in each group. Res culture solution at concentrations of 0, 10, 20, 25, 50, 75, and 100 *μ*mol/L was added to the VSMCs, which incubated at 37°C, 5% CO_2_ for 24 h, and 10 *μ*L CCK8 solution was added to each well, incubated for 30 min at 450 nm, and the absorbance value (A) was detected at 450 nm (cell viability (%) = OD experimental group/OD control group × 100%).

*(2) Cell Model Establishment and Experimental Grouping*. In VSMCs calcification model, cells were inoculated into 6-well plates, and 10 mmol/L *β* glycerol phosphate sodium and 8 mmol/L calcium chloride, ascorbic acid 50 *μ*g/mL, dexamethasone 10 nmol/L, and insulin 1 *μ*mol/L were all induced for 7-10 days [1, 20]. The fluid was changed every two days. In experimental groups, there are control group (DMEM solution with 10% FBS cultured for 7-10 days), AS group (calcified medium cultured for 7-10 days), AS + Res group (10 *μ*mol/L Res preincubated for 40 minutes + calcified medium), and AS +MCC950 group (2 nmol/L + calcified medium).

*(3) Calcification Level of VSMCs Detected by Alizarin Red S Staining*. The VSMCs of different groups were fixed with 4% paraformaldehyde for 15 min at room temperature, stained with 0.2% alizarin red S staining solution which incubated for 30 min, and washed three times with PBS for 1-2 min/time to observe the orange-red calcium nodules.

*(4) Detection of Runx2, NLRP3, Caspase-1, GSDMD, and IL-1β mRNA Expression Levels in VSMCs by RT-qPCR*. VSMCs were collected, and total RNA was extracted using Trizol; the RNA was reverse transcribed into cDNA using a reverse transcription kit, and the target gene was amplified by real-time fluorescence quantitative PCR. The reaction conditions were as follows: PCR amplification system consisted of 2×AceQ Universal SYBR qPCR Master Mix 10.0 *μ*L, primers 0.4 *μ*L (refer to Table 1 for primer sequences), cDNA 1 *μ*L, and dd H_2_O 8.2 *μ*L, making a total system volume of 20 *μ*L (reaction conditions: 95°C predenaturation for 5 min, 95°C annealing for 10 s, 60°C extension for 30 s, with a total of 40 cycles). The relative expression of the target mRNA was calculated by 2 − ΔΔCt with GAPDH as the internal reference.

*(5) Detection of Protein Expression Levels of BMP2, Runx2, NLRP3, Caspase-1, GSDMD, IL-1β, IL-18, and NF-κB p65 in VSMCs by Western Blot*. VSMCs were extracted following stimulation and centrifuged at 4°C for 15 min at 12000 r.min^−1^. The supernatant was removed, and the protein concentration of the samples was quantified by the BCA method [21]. 10% SDS-PAGE gels were prepared for the Western blot procedure, electrophoresis at constant pressure (80v,120 min), membrane transfer at constant current (270 mA, 70 min), 5% skimmed milk powder incubation for 1 h and primary antibody incubation at 4°C overnight, secondary antibody incubation at room temperature for 1 h, ECL solution development, and gel graphic analysis by Image Lab 3.0 system. The grayscale values of each protein band were determined by ImageJ software, and the relative expression was expressed as the ratio of the grayscale values of BMP2, Runx2, NLRP3, Caspase-1, GSDMD, IL-1*β*, IL-18, NF-*κ*B p65, and GAPDH bands.

*(6) Detection of Runx2, NLRP3, Caspase-1, GSDMD, IL-1β, and NF-κB p65 Protein Expression Levels in VSMCs by Immunofluorescence*. VSMCs were seeded in 24-well plates at 2 × 10^3^ cells/well, fixed with 4% paraformaldehyde for 15 min, permeabilized with 0.3% Triton X-100 for 15 min, closed with 5% BSA for 1 h, and incubated with primary antibodies Runx2, NLRP3, Caspase-1, GSDMD, and IL-1*β*, overnight at 4°C. FITC-labeled secondary antibodies were added and incubated for 1 h at room temperature and protected from light, and the nucleus was stained with DAPI for 10 min, sealed with a blocking solution containing antifluorescence quencher, and images were collected and analyzed under a fluorescence microscope.

#### 2.2.2. Animal Experiments

*(1) Animal Experimental Group and Intervention Methods*. 14 male ApoE-/- mice for 6-8 weeks were randomly divided into the control group (Con); in a P53 agonist group (parthenolide, Par group), parthenolide was dissolved in peanut oil; in 7 mice in each group, the con group was fed high-fat chow for 16 weeks, and in the Par group, they were also fed high-fat chow for 16 weeks and injected intraperitoneally with parthenolide at 5 mg/kg for the last 3 weeks [22].

*(2) The Area of Atherosclerotic Plaque in the Carotid Arteries Detected by HE Staining*. After 16 weeks of intervention, the mice were executed by cervical dislocation method; the carotid arteries were isolated, fixed in 4% paraformaldehyde, gradient dehydrated, and embedded; the sections were put in hematoxylin stain for 5 min and put in alcohol gradient dehydration, 1% hydrochloric acid ethanol fractionation, eosin stain for 5-10 s, and neutral gum sealing, and 3 fields were randomly selected under the microscope to observe the pathological changes of the carotid arteries [23].

*(3) Calcification Level of Carotid Arteries Detected by Alizarin Red S Staining*. Paraffin sections were routinely dewaxed and dehydrated, embedded in 95% ethanol, immersed in 0.2% Alizarin Red S staining solution, dipped for 5-10 min, rinsed in distilled water, dehydrated, transparent, and sealed with neutral gum.

*(4) Detection of Runx2, NLRP3, Caspase-1, GSDMD, IL-1β, and NF-κB p65 Protein Expression Levels by Immunofluorescence*. Paraffin sections were routinely dewaxed, dehydrated, washed with distilled water, subjected to antigen repair, and closed with 5% BSA for 30 min; primary antibodies for Runx2, NLRP3, Caspase-1, GSDMD, and IL-1*β* were added overnight at 4°C; FITC-conjugated secondary antibodies were added, incubated for 1 hour at room temperature and protected from light, nuclei stained with DAPI for 10 minutes, and blocked with blocking solution containing antifluorescence quencher. Images were collected under a fluorescence microscope, and the results were analyzed.

## 3. Results

### 3.1. VSMCs Calcification Modeling and Res Intervention

**Figure 1:**
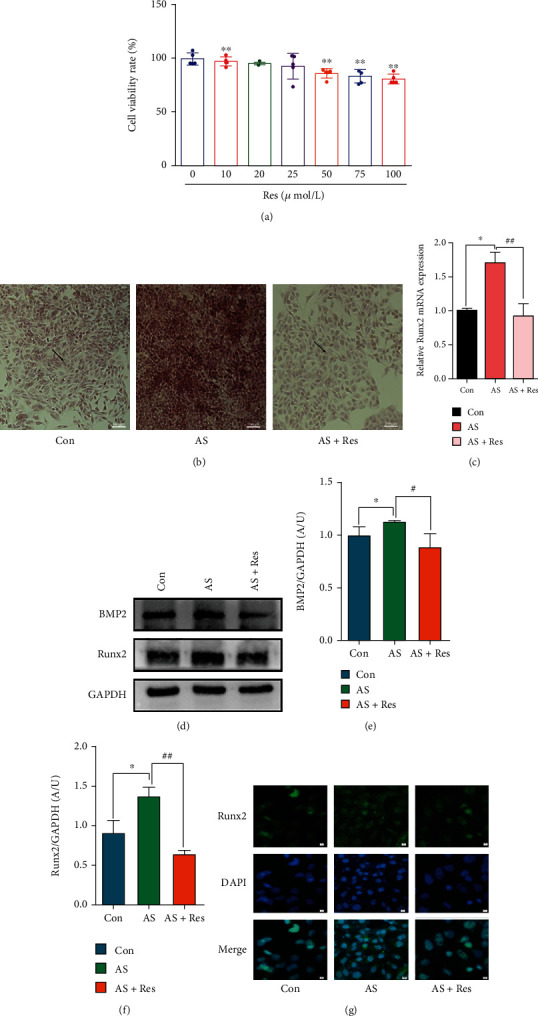
VSMCs (CRL-1999) were treated with Res (10 *μ*M) for the indicated time. (a) Effects of different concentrations of Res on proliferation activity of VSMCs. Data are expressed as mean ± SEM. ^∗∗^*P* < 0.01, vs. 0 *μ*mol/L. (b) Calcium deposition in VSMCs was assessed by alizarin red S staining (positive staining: red; scale bar = 100 *μ*m). (c) The mRNA level of Runx2 was determined by qPCR. Data are expressed as mean ± SEM. ^∗^*P* < 0.05 vs. control group; ^##^*P* < 0.01 vs. AS group. (d) The protein levels of BMP2 and Runx2 were determined by western blotting. (e, f) Quantification of the results shown in (c). Data are expressed as mean ± SEM. ^∗^*P* < 0.05 vs. control group; ^#^*P* < 0.05, ^##^*P* < 0.01 vs. AS group. (g) Representative immunofluorescent staining images for Runx2 (green) (magnification: ×400, scale bar = 20 *μ*m).

To investigate the optimal concentration of Res for VSMCs calcification modeling and intervention, VSMCs were stimulated with different concentrations of Res, and the cell proliferation activity was measured by the CCK8 kit after 24 h. The results showed that the cell proliferation activity decreased gradually with an increase in concentration after the Res effect concentration was higher than 10 *μ*mol/L (Figure 1(a)). Thus, 10 *μ*mol/L was selected as the optimal stimulation concentration of Res for this study. After 7-10 days of induction by stimulation of VSMCs with calcifying medium, intracellular calcium salt deposition was detected by alizarin red S staining. The results showed that compared with the control group, the AS group had an increase in orange-red calcium salt particles and visible nodules (Figure 1(b)). However, the administration of Res intervention resulted in decreased calcium salts in VSMCs and attenuated calcified nodules. The qPCR results showed that the mRNA level of Runx2 was significantly increased in the AS group and attenuated in the AS + Res group (Figure 1(c)). The western blot results showed that BMP2 and Runx2 protein levels increased in the AS group, and both significantly attenuated in the AS + Res group (Figures 1(d)–1(f)). The immunofluorescence results showed that Runx2 expression levels increased in the AS group and attenuated in the AS + Res group (Figure 1(g)).

### 3.2. Effects of Res on NLRP3 and Related Proteins

**Figure 2:**
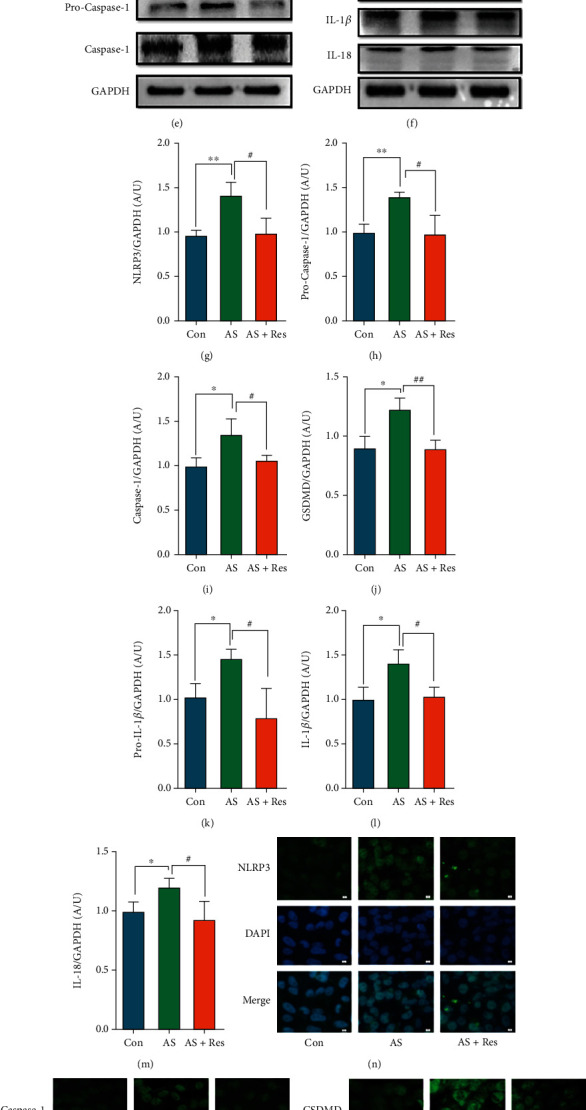
VSMCs (CRL-1999) were treated with Res (10 *μ*M) for indicated time. (a–d) The mRNA levels of NLRP3 and caspase-1, GSDMD, and IL-1*β* were determined by qPCR. Data are expressed as mean ± SEM. ^∗∗^*P* < 0.01 vs. control group; ^#^*P* < 0.05, ^##^*P* < 0.01 vs. AS group. (e, f) The protein levels of NLRP3 and pro-caspase-1, caspase-1, GSDMD, and pro-IL-1*β*, IL-1*β*, and IL-18 were determined by western blotting. (g–m) Quantification of the results shown in (e, f). Data are expressed as mean ± SEM. ^∗^*P* < 0.05, ^∗∗^*P* < 0.01 vs. control group; ^#^*P* < 0.05, ^##^*P* < 0.01 vs. AS group. (n–q) Representative immunofluorescent staining images for NLRP3 and caspase-1, GSDMD, and IL-1*β* (green) (magnification: ×400, scale bar = 20 *μ*m).

Compared with the control group, the mRNA levels of NLRP3 and caspase-1, GSDMD, and IL-1*β* were significantly increased in the AS group and decreased in the AS + Res group (Figures 2(a)–2(d)). Western blot results showed that the levels of NLRP3 and pro-caspase-1 and caspase-1, GSDMD, Pro-IL-1*β*, IL-1*β*, and IL-18 proteins were significantly enhanced in the AS group and descended in the AS + Res group (Figures 2(e)–2(m)). Immunofluorescence results showed that NLRP3 and caspase-1 proteins ascended significantly in the AS group and declined in the AS + Res group (Figures 2(n)–2(q)).

### 3.3. Effects of Res on Transcription Factor NF-*κ*B p65

**Figure 3:**
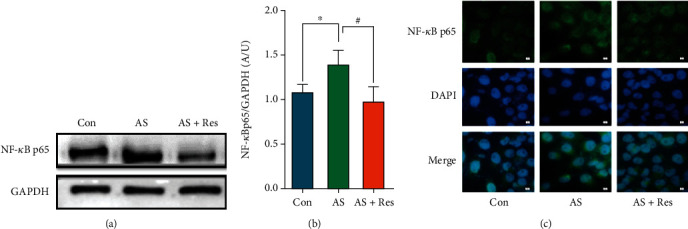
VSMCs (CRL-1999) were treated with Res (10 *μ*m) for the indicated time. (a) The protein level of NF-*κ*B p65 was determined by western blotting. (b) Quantification of the results shown in (a). Data are expressed as mean ± SEM. ^∗^*P* < 0.05 vs. control group; #*P* < 0.05 vs. AS group. (c) Representative immunofluorescent staining images for NF-*κ*B p65 (green) (magnification: ×400, scale bar = 20 *μ*m).

Western blot results showed a significant increase in NF-*κ*B p65 protein levels in the AS group and a significant attenuation in the AS + Res group compared to the control group (Figures 3(a) and 3(b)). Immunofluorescence results showed an increase in NF-*κ*B p65 protein and a downtrend in the AS + Res group (Figure 3(c)).

### 3.4. Effects of MCC950 on Intracellular Calcium Salt Deposition

**Figure 4:**
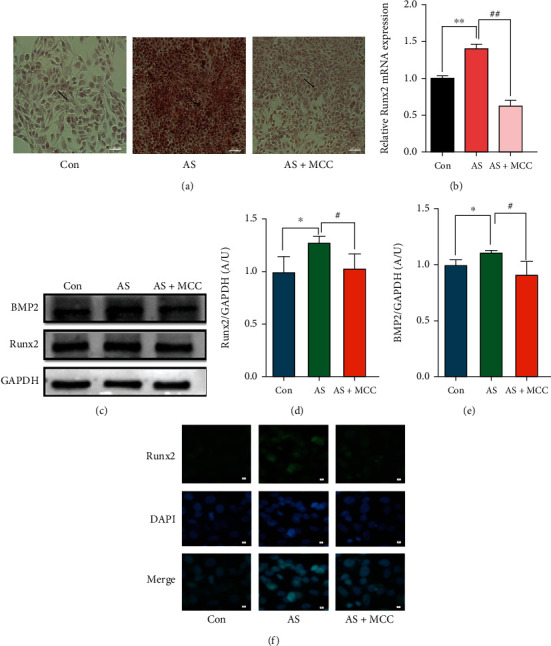
VSMCs (CRL-1999) were treated with NLRP3 inhibitor (MCC950; 2nM) for indicated time. (a) Calcium deposition in VSMCs was assessed by alizarin red S staining (positive staining: red; scale bar = 100 *μ*m). (b) The mRNA levels of Runx2 were determined by qPCR. Data are expressed as mean ± SEM. ^∗∗^*P* < 0.01 vs. control group; ^##^*P* < 0.01 vs. AS group. (c) The protein levels of BMP2 and Runx2 were determined by western blotting. (d, e) Quantification of the results shown in (c). Data are expressed as mean ± SEM. ^∗^*P* < 0.05 vs. control group; ^#^*P* < 0.05 vs. AS group. (f) Representative immunofluorescent staining images for Runx2 (green) (magnification: ×400, scale bar = 20 *μ*m).

Compared with the control group, the AS group showed significant calcified nodules. Compared with the AS group, the effect of NLRP3 inflammasome inhibitor MCC950 on VSMCs showed a significant decrease (Figure 4(a)). The Runx2 mRNA level was significantly increased in the AS group and decreased after MCC950 intervention (Figure 4(b)). The western blot results showed that the BMP2 and Runx2 protein levels were significantly increased in the AS group but decreased in the AS + MCC group (Figures 4(c)–4(e)). Immunofluorescence revealed that Runx2 protein levels were elevated in the AS group, while significantly downregulated in the AS + MCC group (Figure 4(f)).

### 3.5. Effects of MCC950 on NLRP3 and Related Proteins

**Figure 5:**
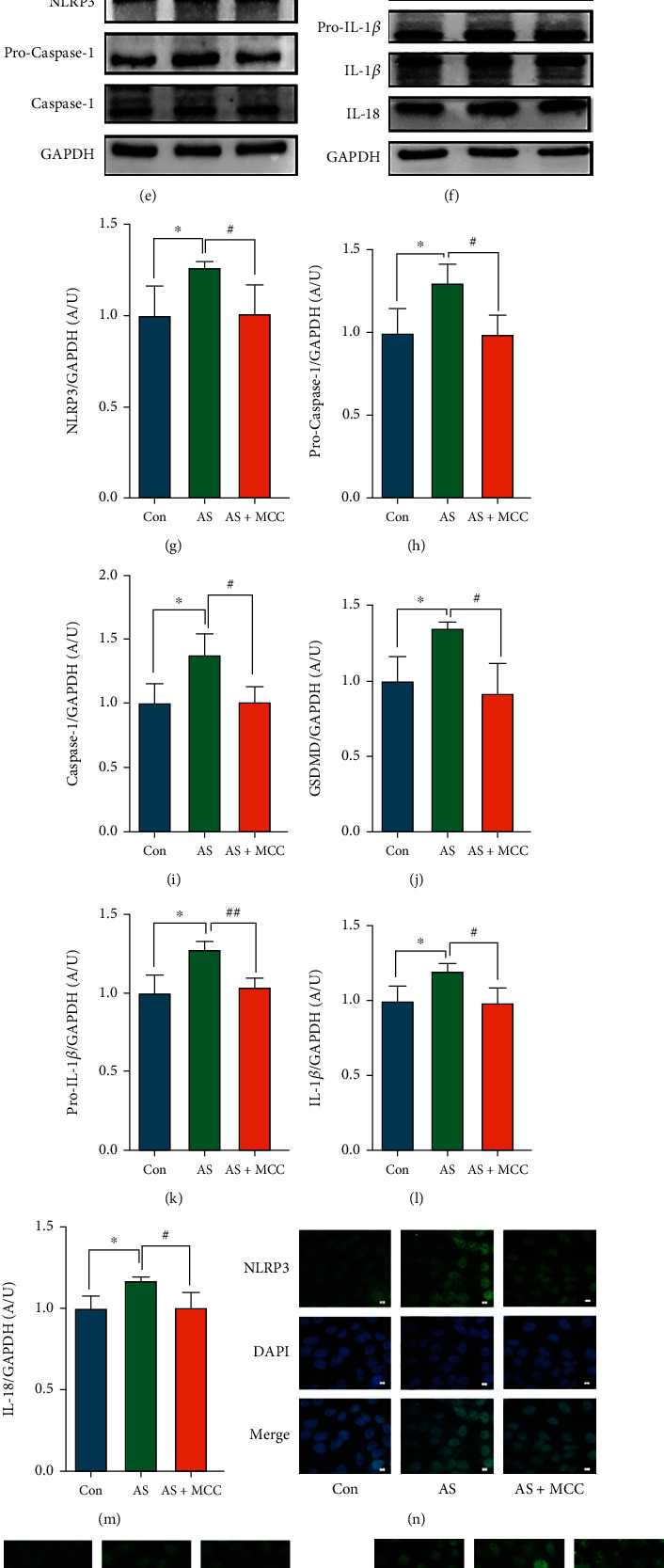
VSMCs (CRL-1999) were treated with NLRP3 inhibitor (MCC950: 2nM) for indicated time. (a–d) The mRNA levels of NLRP3 and caspase-1, GSDMD, and IL-1*β* were determined by qPCR. Data are expressed as mean ± SEM. ^∗∗^*P* < 0.01 vs. control group; ^#^*P* < 0.05, ^##^*P* < 0.01 vs. AS group. (e, f) The protein levels of NLRP3 and pro-caspase-1, caspase-1, GSDMD, and pro-IL-1*β*, IL-1*β*, and IL-18 were determined by western blotting. (g–m) Quantification of the results shown in (e, f). Data are expressed as mean ± SEM. ^∗^*P* < 0.05 vs. control group; ^#^*P* < 0.05 vs. AS group. (n–q) Representative immunofluorescent staining images for NLRP3 and caspase-1, GSDMD, and IL-1*β* (green) (magnification: ×400, scale bar = 20 *μ*m).

Compared with the control group, the NLRP3 and related protein levels were significantly increased in the AS group. After administration of MCC950, the NLRP3 and related protein levels were attenuated (Figures 5(e)–5(m)). These results indicated that MCC950 had an inhibitory effect on NLRP3 and related proteins. The immunofluorescence results showed that NLRP3, caspase-1, GSDMD, and IL-1*β* protein expression were enhanced in the AS group, while declined in the AS + MCC group (Figures 5(n)–5(q)).

### 3.6. Effects of MCC950 on Transcription Factor NF-*κ*B p65

**Figure 6:**
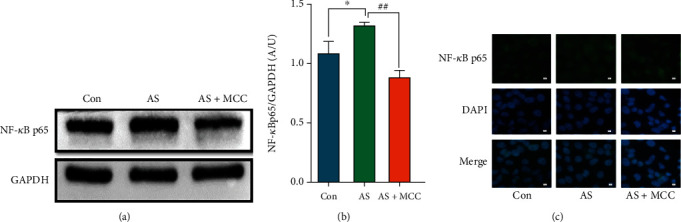
VSMCs (CRL-1999) were treated with NLRP3 inhibitor (MCC950: 2 nM) for the indicated time. (a) The protein levels of NF-*κ*B p65 were determined by western blotting. (b) Quantification of the results shown in (a). Data are expressed as mean ± SEM. ^∗^*P* < 0.05 vs. control group; *^##^P* < 0.01 vs. AS group. (c) Representative immunofluorescent staining images for NF-*κ*B p65 (green) (magnification: ×400, scale bar = 20 *μ*m).

Compared to the control group, the western blot results showed that the NF-*κ*B p65protein expression was significantly increased in the AS group and significantly attenuated in the AS + MCC group (Figures 6(a) and 6(b)). The immunofluorescence results showed that NF-*κ*B p65 protein expression ascended in the AS group, while descended in the AS + MCC group (Figure 6(c)).

### 3.7. P53 Agonists Exacerbated Plaque Pathology and Aggravated Calcium Salt Deposition Levels in ApoE-/- Mice

**Figure 7:**
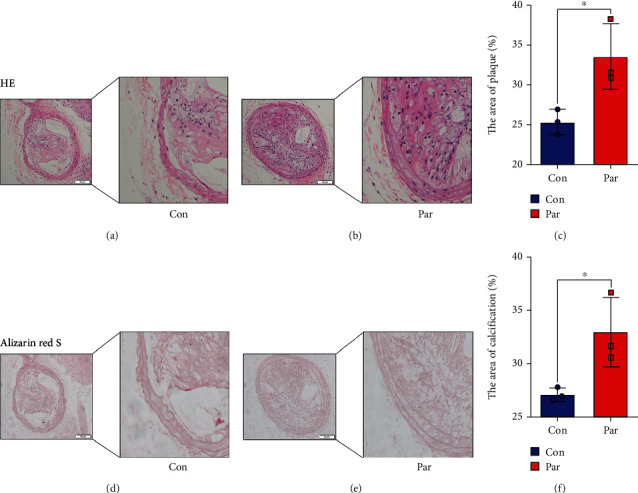
The carotid arteries of ApoE-/-male mice. (a, b) Histopathologic assessment of the vascular of ApoE-/-mice after feed high fat feed intraperitoneal and injection of Par (5mg/kg) for 16 weeks. Shown are representative H&E-stained sections of carotid arteries from two independent experiments (*n* = 7; positive staining: brown to black; scale bar = 100 *μ*m). (c) Quantification of the results shown in (a, b). Data are expressed as mean ± SEM. ^∗^*P* < 0.05 vs. control group. (d, e) Calcification was assessed by alizarin red staining (positive staining: red; scale bar = 100 *μ*m). (f) Quantification of the results shown in (d, e). Data are expressed as mean ± SEM. ^∗^*P* < 0.05 vs. control group.

Compared with the control group, the Par group showed a significant vascular hyperplasia by HE staining. Additionally, the level of calcium salt deposition was significantly higher in the Par group compared to the control group (Figures 7(a)–7(d)).

### 3.8. P53 Agonists Increased the Expression Levels of BMP2, Runx2, NLRP3, Caspase-1, GSDMD, IL-1*β*, and NF-*κ*B p65

**Figure 8:**
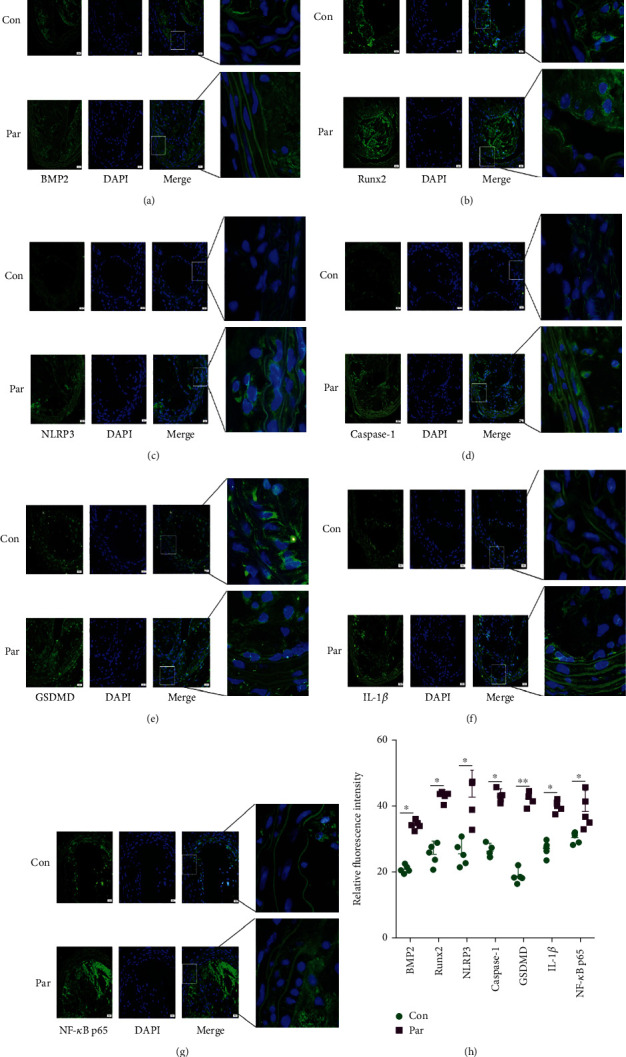
The carotid arteries of ApoE-/-male mice. (a–g) Representative immunofluorescent staining images for BMP2 and Runx2, NLRP3 and Caspase-1, GSDMD and IL-1*β*, and NF-*κ*B p65 (green) (magnification: ×400, scale bar = 20 *μ*m). (h) Quantification of the results shown in (a–g). Data are expressed as mean ± SEM. ^∗^*P* < 0.05, ^∗∗^*P* < 0.01 vs. control group.

The immunofluorescence results showed that the expression of BMP2 and Runx2, NLRP3, Caspase-1, GSDMD, IL-1*β*, and NF-*κ*B p65 increased in the Par group compared to the control group. These findings indicated that the P53 agonist Par tends to promote the calcification formation and worsen the plaque lesions (Figures 8(a)–8(g)).

## 4. Discussion

Vascular calcification is the end-stage manifestation of AS and is a risk factor for cardiovascular disease [24–26]. In this study, we constructed a calcification model in vitro and found the appearance of calcified nodules. The expression of calcification indicators BMP2 and Runx2 increased while inflammatory indicators NLRP3 and IL-1*β* were elevated, and the expression of pyroptosis indicators GSDMD, caspase-1, and transcription factor NF-*κ*B p65 were also enhanced. Res intervention resulted in a reduction in the calcified nodules and a decrease in the expression of calcification indicators BMP2 and Runx2. Additionally, the expression of inflammatory indicators, NLRP3 and IL-1*β*, and pyroptosis indicators, GSDMD and NF-*κ*B p65, all declined. The expression of NLRP3, GSDMD, NF-*κ*B p65, and other inflammatory indicators also descended, while the MCC950 inhibited NLRP3 and reduced the expression of these indicators. In vivo experiments, Par had a provascular membrane proliferation effect on AS lesions, which increased the calcification indexes BMP2, Runx2, inflammatory index NLRP3, and pyroptosis index GSDMD. Therefore, we found that P53 agonists exacerbated the AS lesions by promoting pyroptosis and inflammatory responses, providing explanatory mechanisms for Res and MCC950 to alleviate AS. We have included a diagram of the mechanisms associated with the relevant drugs and VSMCs.

VSMCs converted from a contractile phenotype to a secretory phenotype. Runx2, a key regulator of osteoblastic cells, mediates calcification of VSMCs by regulating BMP2 expression [3, 27, 28]. Alizarin red S staining showed that Res reduced calcified nodules in VSMCs and decreased calcium salt deposition within VSMCs. This is consistent with the previously reported findings that Res inhibits the development of calcification in hVSMCs induced by high calcium and phosphorus conditions [29]. Res was also found to reduce blood pressure and improve cardiac function [30].

VSMCs death activates the inflammatory response, and inflammatory factors trigger the inflammatory cascade response, leading to a large number of inflammatory cell aggregation, which promotes AS plaque enlargement, and fibrous cap shedding, which induces plaque rupture and bleeding [31, 32]. In addition, VSMCs proliferation promotes the process of AS development.

When VSMCs are exposed to various stimuli, oxidative stress, mitochondrial dysfunction, and lysosomal disruption, large amounts of ROS are generated, which promote the activation of inflammatory vesicles and induce pyroptosis [33, 34]. In this study, we found that the mRNA and protein expression of NLRP3, caspase-1, GSDMD, and IL-1*β* were upregulated after calcification in VSMCs, indicating the presence of inflammatory responses and the occurrence of pyroptosis in VSMCs. The mRNA and protein expression of NLRP3, caspase-1, GSDMD, and IL-1*β* were increased after calcification of VSMCs, indicating the presence of inflammatory response and the occurrence of pyroptosis in VSMCs. Related indicators were significantly decreased after Res treatment. The expression of BMP2 and Runx2 was decreased, which demonstrated that Res inhibited the occurrence of the above processes and suppressed the transformation of VSMCs to osteoblast-like cells. These results are consistent with the experiment that Res inhibits liver fibrosis by regulating the NLRP3/caspase-1/IL-1*β* signaling pathway [35], and the intervention of SD rat high-fat model reduces the expression of NF-*κ*B p65, IL-1*β*, IL-18, NLRP3, and other proteins [29]. These findings suggested that Res may inhibit calcification by regulating the NF-*κ*B/NLRP3/IL-1*β* pathway, thereby reducing Runx2 levels, VSMCs proliferation, differentiation, and alleviating AS lesions.

A related study found that MCC950 alleviated macrophage inflammation and pyroptosis by inhibiting the assembly and activation phase of NLRP3 inflammasome, which reduced plaque area in ApoE-/- mice, and a study showed that inflammatory released NLRP3 inflammasome and IL-18 and IL-1*β* [17]. The receptor proteins of inflammasomes NLRP3, NLRP1, and AIM2 are activated to bind to ASC and activate pro-caspase-1 to be caspase-1 and pro-IL-1*β* and pro-IL-18 into proinflammatory factors IL-1*β* and IL-18 through protein hydrolysis, inducing pyroptosis [36, 37]. In this study, we found that MCC950 interfered with VSMCs to reduce calcified nodules. The mRNA and protein levels of NLRP3, GSDMD, IL-1*β*, and Runx2 were found to attenuate calcification by inhibiting NLRP3 inflammasome in VSMCs to alleviate inflammation and pyroptosis and decreased NLRP3 and Caspase-1 by inhibiting NF-*κ*B pathway [38]. We proved that MCC950 also inhibited NF-*κ*B p65, which regulates downstream proteins. These findings demonstrated that MCC950 can inhibit inflammatory response with pyroptosis and calcification through the NF-*κ*B/NLRP3/IL-1*β* pathway.

MCC950 is an inhibitor of the NLRP3 inflammasome, which was responsible for releasing inflammatory substances such as IL-18 and IL-1*β* [39]. Res-induced apoptosis inhibited the NF-*κB* pathway and the activation of NLRP3 inflammasomes, resulting in the downregulated expression of IL-1*β*, IL-18, and IL-6 [40, 41]. In the study, MCC950 was used as a positive control drug to compare with Res. The results showed that Res had a better effect on NF-*κ*B, NLRP3, IL-1*β*, and IL-18 than MCC950. Therefore, we believed that Res and MCC950 have the potential to suppress calcification, the inflammatory response, and pyroptosis by regulating the NF-*κ*B/NLRP3/IL-1*β* axis, which was related to the activation of the NLRP3 inflammasome. This mechanism could be important to using Res and MCC950 as a preventative measure and as a therapy for AS to improve prognosis.

The previous study reported that the levels of P53 significantly increased in ApoE-/- mice fed a high-fat diet [42]. Furthermore, the P53 expression was enhanced by LincRNA-p21 to regulate neointima, VSMCs proliferation, apoptosis, and the formation of AS [43, 44]. We used the P53 agonist Par to investigate the phenomenon in the AS group. Our findings demonstrated that Par promoted AS plaque lesions and vascular membrane proliferation disorders and increased calcium salt deposition. These findings indicated that the enhanced P53 expression increased plaque area. The immunofluorescence analysis revealed that the expression of Runx2 and BMP2, NLRP3 and IL-1*β*, GSDMD and Caspase-1, and NF-*κ*B p65 is slightly upregulated in the control group. These findings suggested that high lipid promotes the related protein expression through inflammation and pyroptosis. However, all these factors were significantly elevated in the Par group. Therefore, we found that the increased P53 expression could intensify the inflammatory response and pyroptosis, promote the expression of Runx2, and increase the AS plaque area.

## 5. Conclusion

**Figure 9:**
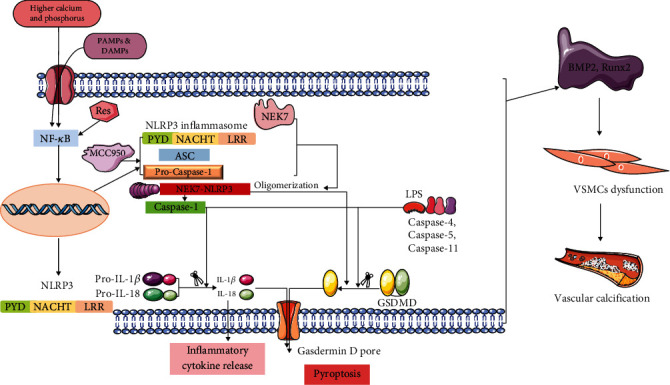
A graphic illustration of the mechanism action of Res, MCC950, and P53 in AS-associated VC.

Our study showed that the calcification medium successfully induced the AS model, which expressed the increased related indexes. However, we found that the intervention of Res and MCC950 could inhibit the calcification indexes Runx2 and BMP2 by suppressing the transcription factor NF-*κ*B p65, inflammatory index NLRP3 and IL-1*β*, pyroptosis Caspase-1, and GSDMD. Furthermore, Res and MCC950 were found to be effective in alleviating calcification of VSMCs, with Res having a better effect than MCC950. On the other hand, Par promoted the AS plaque formation and aggravated the AS lesions by significantly elevating the related index. We demonstrated that the NF-*κ*B/NLRP3/IL-1*β* pathway was affected by P53, Res, and MCC950, which can regulate the inflammatory response and pyroptosis. The associated signaling pathway is shown in Figure 9. Moreover, P53 also inhibits calcification, inflammatory reaction, and pyroptosis, thus alleviating the AS plaque. Therefore, Res and P53-related agents can be promising drugs for the clinical treatment of AS disease. In conclusion, Res, MCC950, and P53 agonists merit additional investigation as a potential treatment for AS.

## Figures and Tables

**Table 1:** Primer sequences.

Primer	Sequence	Length (bp)
Runx2	F: 5′-GAGTGGACGAGGCAAGAGTT-3′	195
	R: 3′-GGATGAGGAATGCGCCCTAA-5′	

NLRP3	F: 5′-ATGTGGGGGAGAATGCCTTG-3′	199
	R: 3′-TTGTCTCCGAGAGTGTTGCC-5′	

Caspase-1	F: 5′-TTTCCGCAAGGTTCGATTTTCA-3′	53
	R: 5′-GGCATCTGCGCTCTACCATC-3′	

IL-1*β*	F: 5′-AGCTACGAATCTCCGACCAC-3′	186
	R: 3′-CGTTATCCCATGTGTCGAAGAA-5′	

GSDMD	F: 5′-GCCTCCACAACTTCCTGACAGATG-3′	86
	R: 3′-GGTCTCCACCTCTGCCCGTAG-5′	

GAPDH	F: 5′-GAAGACGGGCGGAGAGAAAC-3′	159
	R: 3′-GCCCAATACGACCAAATCCGT-5′	

## Data Availability

The data used to support the findings of this study are available from the corresponding authors upon request.
